# Q-Learning Based Fair and Efficient Coexistence of LTE in Unlicensed Band

**DOI:** 10.3390/s19132875

**Published:** 2019-06-28

**Authors:** Rojeena Bajracharya, Rakesh Shrestha, Sung Won Kim

**Affiliations:** 1 Information and Communication Engineering, Yeungnam University, Gyeongsan 38541, Korea; 2 Yonsei Institute of Convergence Technology, Yonsei University, Incheon 21983, Korea

**Keywords:** efficient, fair, IoT, multimedia traffic, LTE-U, spectrum, Q-learning, Wi-Fi

## Abstract

The increased demand for spectrum resources for multimedia communications and a limited licensed spectrum have led to widespread concern regarding the operation of long term evolution (LTE) in the unlicensed (LTE-U) band for internet of things (IoT) systems. Because Wi-Fi and LTE are diverse with dissimilar physical and link layer configurations, several solutions to achieve an efficient and fair coexistence have been proposed. Most of the proposed solutions facilitate a fair coexistence through a discontinuous transmission using a duty cycling or contention mechanism and an efficient coexistence through a clean channel selection. However, they are constrained only by fairness or efficient coexistence but not both. Herein, we propose joint adaptive duty cycling (ADC) and dynamic channel switch (DCS) mechanisms. The ADC mechanism supports a fair channel access opportunity by muting certain numbers of subframes for Wi-Fi users whereas the DCS mechanism offers more access opportunities for LTE-U and Wi-Fi users by preventing LTE-U users from occupying a crowded channel for a longer time. To support these mechanisms in a dynamic environment, LTE-U for IoT applications is enhanced using Q-learning techniques for an automatic selection of the appropriate combination of muting period and channel. Simulation results show the fair and efficient coexistence achieved from using the proposed mechanism.

## 1. Introduction

**Figure 1:**
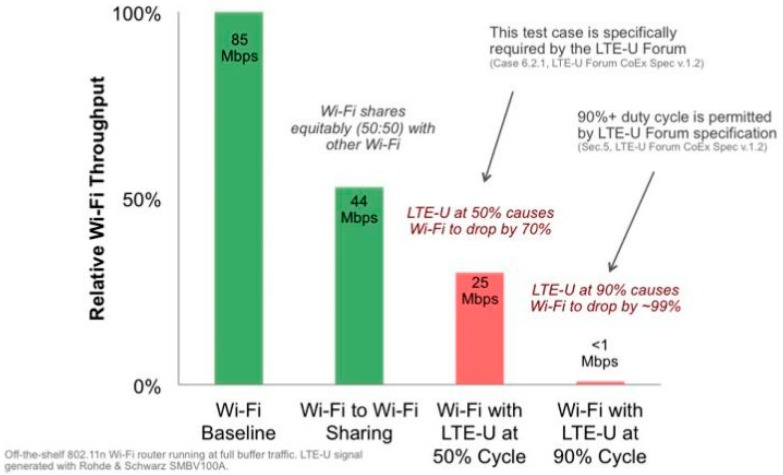
CableLabs experiment on LTE-U [7].

**Figure 2:**
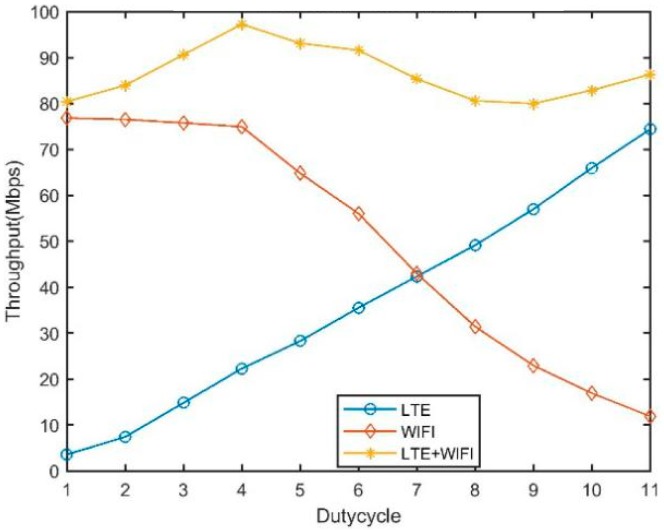
Preliminary simulation results of duty cycle LTE-U.

An exponential increase in demand for wireless multimedia data and the limited nature of a licensed spectrum for cellular networks have inspired the use of unlicensed bands for long term evolution (LTE) in internet of things (IoT). In an unlicensed spectrum of below 6 GHz, a large part of spectrum, approximately 600 MHz [1], is globally available for various purposes. LTE uses this unlicensed band to offload multimedia traffic through either downlink-only or both downlink and uplink approaches in IoT systems. Although, the use of LTE in an unlicensed band enhances the capacity and achieves a seamless user experience, a few issues in allowing different networks to operate in a mutually shared spectrum need to be considered. One important issue is the coordination and management of an interference among the different coexisting technologies [2,3]. A Wi-Fi system utilizes the carrier sense multiple access (CSMA) protocol to coexist harmoniously with various other Wi-Fi systems, whereas LTE uses continuous traffic generation with the smallest time gaps even in the absence of data traffic. Considering these operating characteristics in both systems, Wi-Fi seems to have a minimal opportunity to use the channel compared with LTE under a coexistence scenario, resulting in a performance degradation for Wi-Fi [4]. In the literature, some solutions such as license assisted access (LAA) [5] and LTE-U [6] have been proposed for IoT systems. LAA is based on the cellular industry’s standard body, the Third-Generation Partnership Project (3GPP), and its coexistence processes follows the same path used in Wi-Fi, i.e., listen before talk (LBT). By contrast, LTE-U is built on proprietary technology established by the LTE-U forum, which takes a completely different approach. A small group of companies such as Ericsson, Qualcomm, Verizon, and Samsung have been developing LTE-U through a closed process. LTE-U applies a carrier ON/OFF switch which duty cycles the LTE transmissions. It switches to ON mode to transmit the signal for a certain amount of time and then it switches to OFF mode so that Wi-Fi can access the channel. Most of the proposed fair coexistence mechanisms for LTE-U largely suffer from a loss of spectrum resource efficiency, while maintaining the airtime and throughput fairness with Wi-Fi [7,8,9,10]. The experiment conducted by CableLabs [7], shown in Figure 1, which demonstrated the fairness conceived by LTE-U over a proportionate airtime, has a disproportionately negative impact on the Wi-Fi performance, i.e., a 50% duty cycle of LTE causes a Wi-Fi performance decrease of 70%. As a result, LTE-U can be ON for only 35% of the cycle to maintain 50% Wi-Fi throughput. Similar to the authors in [8], our initial simulation results demonstrate that a change in duty cycle affects both LTE-U and Wi-Fi under a coexistence scenario. We can see that, to achieve a fair coexistence, the maximum achievable throughput of the network is significantly reduced (18%) and vice versa, as shown in Figure 2. This occurs because of the unbalanced physical and link layer parameters between these two technologies. In the case of Wi-Fi, most of the channel resources are wasted during contention, thereby requiring significant radio resources to maintain equal throughput as compared to LTE-U. Because both technologies have an equal right to use the unlicensed band, Wi-Fi can be regarded as the least efficient technology compared to the schedule-based LTE-U technology in terms of spectrum utilization [11,12]. Hence, enabling a fairness measure among Wi-Fi and LTE-U may lead to an underutilization of the wireless spectrum resources compared to the exploitation of the entire unlicensed band. Similarly, in the context of increasing the network efficiency, the studies described in [13,14,15,16,17] have contributed to an increase in network efficiency by simply scarifying the fairness measure under a coexistence scenario. Thus, fairness and efficiency are the two critical and conflicting criteria in spectrum resource management under a Wi-Fi and LTE-U coexistence scenario.

The present study attempted to reduce the above-mentioned conflicts by taking advantage of the existence of multiple channels in the 5-GHz unlicensed spectrum. The 5-GHz band offers multiple non-overlapping 20/40/80/160 MHz channels. Because all Wi-Fi APs are provisioned to operate in different channels based on the received power measurement, the traffic load on each available channel will be diverse. Therefore, to increase their throughput performance, choosing the least congested channel for the operation of LTE-U with Wi-Fi will be highly advantageous for LTE-U and Wi-Fi individually. The best channel selection approaches are greatly subsidized to increase the effectiveness of the network but not the fairness. Hence, we designed a combination of adaptive duty cycling (ADC) and dynamic channel switch (DCS) mechanisms for the network to access a channel under a dynamic traffic load scenario. The ADC mechanism supports a fair channel access opportunity by reserving a certain number of subframes for the operation of Wi-Fi Stations (STAs). In contrast, the DCS mechanism offers more access opportunities for LTE-U users by avoiding most crowed channels for their operation. When considering futuristic wireless applications, the user-demand will vary across a wider spectrum. Such requirements cannot be satisfied by assigning fixed resources. Hence, the realization of LTE-U collocated with Wi-Fi under a dynamic environment is a prerequisite. Thus, LTE-U is enhanced using Q-learning techniques for an autonomous selection of the appropriate combination of best duty cycles in various channels through iterations of the learning process. This process escalates the spectrum efficiency of the network while assuring the fairness among these LTE and Wi-Fi systems. Meanwhile, to simulate the proposed scenario, a system-level simulation program for a Wi-Fi and LTE-U system was built and utilized. The analysis of the simulation results demonstrates that the proposed algorithm enhances the network performance while sustaining the fairness among Wi-Fi and LTE-U systems. The main contributions of this study are as follows:

- Description and analysis of collocated LTE-U and Wi-Fi system;
- A Q-learning mechanism used for an ideal and autonomous selection of an LTE-U operational channel muting duration toward fair and efficient spectrum sharing under a dynamic environment;
- A performance evaluation of the proposed Wi-Fi and LTE-U coexistence mechanism with pre-existing coexistence solutions, i.e., duty cycle (DC) only and channel occupancy time (COT) based channel selection.

The remainder of this paper is organized as follows. Section 2 details and reviews some recent related studies. Section 3 discusses the proposed LTE and Wi-Fi coexistence mechanism. Section 4 describes the simulation results and provides a detailed evaluation of its performance. Section 5 provides some concluding remarks.

## 2. Related Studies

In recent years, extensive research has been conducted to assure fair coexistence between LTE-U and Wi-Fi networks. Some countries have prohibited a continuous signal transmission and bind limits on the maximal duration of a transmission burst in an unlicensed spectrum, and as a result, carrier sense adaptive transmission (CSAT) [18,19] was introduced during the early deployment of LTE in the unlicensed band. CSAT permits an LTE-U network to share an unlicensed channel with a Wi-Fi network through time division multiplexing. To implement CSAT, the existing almost-blank subframes (ABS) framework (i.e., defined in 3GPP Release 10 of LTE) was initially considered in [20]. The ABS reserves a group of LTE subframes, during which the macro UEs are partly muted (data, control, or reference symbols), allowing the UEs in pico-base stations (BSs) to be assisted with a lower interference [21]. An LTE silence period occurs during these gaps, allowing the Wi-Fi system to access the channel. Wi-Fi uses these gaps for transmission and must end its transmission whenever the communication is resumed by LTE-U. However, synchronization signals and control information are still present in ABS, which may influence the Wi-Fi transmissions and carrier sensing, as discussed in [22]. To overcome this issue, the LTE-U forum [2] adopted the carrier aggregation feature of MAC channel element activation and deactivation, which is compatible with the Rel. 10/11/12 LTE PHY/MAC standards. Many studies based on LTE-U have since been proposed. Rupasinghe et al. [9] introduced the LTE time division duplex (TDD) configuration with different numbers of uplink and downlink frames for LTE-U. However, their configuration consists of numerous uplink slots, making it inefficient under a real scenario. Under such a real scenario, more downlink frames are expected than uplink frames in any network. Cano and Leith in [19] proposed a duty-cycle mechanism for LTE-U that selects the suitable probability to access the channel and transmission duration. This ensures proportional fairness among LTE-U and Wi-Fi nodes. Almeida et al. [21] showed that, without a proper ABS assignment, the throughput of a Wi-Fi network under the coexistence of an LTE-U network can be seriously degraded, and similar concerns were reported in [22]. Likewise, in their technical report, the 3GPP Workgroup listed LBT [22,23,24,25] for LTE LAA for nations such as Japan and European countries. The application of LBT potentially enhances the coexistence, such as with Wi-Fi, through a clear channel assessment. Some analysis and performance tests have been reported toward the fairness under such a scenario [26,27]. However, a duty-cycle based approach still utilizes resources more tightly and with no modifications of the LTE standard [19].

In the context of increased network efficiency, approaches have been proposed [13,14,15] to increase the LTE-U efficiency through the selection of most of the idle channels. Similarly, the authors in [28] proposed a multichannel coexistence approach to increase the spectrum efficiency through which LTE-U uses all available channels simultaneously. However, the realization of such an approach requires a vast change in the preexisting system hardware.

## 3. LTE-U Coexistence Mechanism

There is approximately 600 MHz of spectrum below the 6-GHz unlicensed bands, which can be further subdivided into multiple channels of the same or different bandwidths. Because Wi-Fi is provisioned to connect to a channel with less interference, the traffic load offered by Wi-Fi in such channels will differ. Therefore, simply considering the single best unlicensed channel for studying a coexistence scenario does not meet the requirements of a practical environment. This paper considers the number of different channels in the unlicensed band and creates a utility function by considering the network efficiency and fairness factor. The duty cycle of LTE-U is adaptively manipulated using the Q-learning mechanism to increase the performance of the systems, thereby allowing LTE-U to occupy various unlicensed bands for a suitable duration.

### 3.1. Deployment Environment

**Figure 3:**
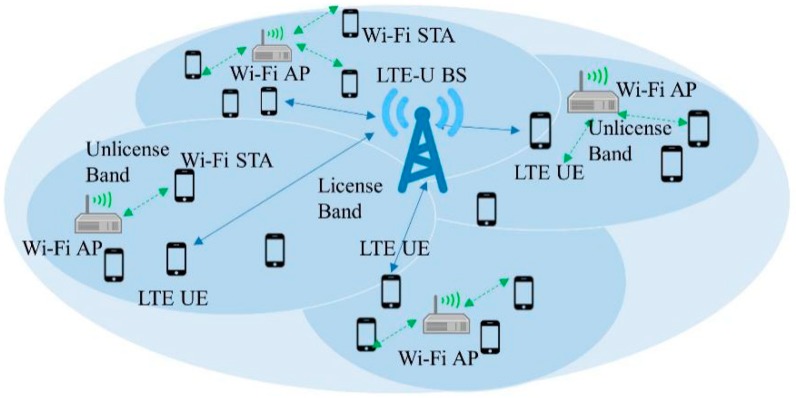
LTE-U deployment scenario.

We consider the deployment scenario shown in Figure 3, in which LTE-U BS consists of multiple Wi-Fi access points (APs), which operate simultaneously in the unlicensed channel. LTE-U BS and each Wi-Fi AP consists of *M* number of LTE-U user equipment (UE) and *N* Wi-Fi stations (STAs) respectively, which are arbitrarily distributed within the coverage area of the cell. TDD LTE is taken into consideration, and it is assumed that LTE-U BS and UEs are synchronized with each other for the entire duration. In the 5-GHz band, a total of *K* unlicensed channels is accessible, with one AP and a set of *N_k_* STAs active in each unlicensed channel *k*. The LTE-U system communicates over the LTE air interface. The BS informs the UEs regarding the use of a channel for transmission over the licensed band. There are *B* resource blocks (RBs) accessible for transmission during each transmission time frame (TTF), where *B* is the bandwidth of an unlicensed channel. The total aggregated throughput served by LTE-U BS in channel *k* during a single TTF can thus be mathematically assessed as follows:

(1) $$R_{k}^{LTE-U}=\overset{M_{k}}{\underset{m_{k}=1}{\sum }}\frac{B}{M_{k}}\;*SINR_{m_{k}}*\left(1-\Theta_{k}\right)$$

where $M_{k}$ represents the total number of LTE-U UEs assisted by the LTE-U BS exploiting the supplemental downlink capacity in channel *k*, $SINR_{m_{k}}\;$ is the signal-to-noise and interference ratio perceived by the *m*th UE when downlink data are conveyed on the *k*th channel, and Θ  is the portion of time related with the idle periods imposed by the LTE-U strategy on the *k*th channel. In addition, *SINR_m_* delivers the spectral efficiency, which relies on the propagation environments between the *m*th UE and the LTE-U BS and the interference produced by other cells using the *k*th channel.

In a Wi-Fi network, all STAs contend for channel access by means of a carrier sensing scheme called a distributed coordination function (DCF) protocol. In a DCF, the system throughput of the Wi-Fi network is determined based on the number of contending Wi-Fi STAs. Let $P_{k}^{tr}\;$ represent the probability of at least one transmission signal being present in a time slot, and $P_{k}^{s}\;$ denote the probability of a successful transmission on a channel, which can be mathematically formulated as

(2) $$P_{k}^{tr}=1-{\left(1-\tau_{k}\right)}^{N_{k}}$$

(3) $$P_{k}^{s}\;=\;\frac{N_{k}\tau_{k}{\left(1-\tau_{k}\right)}^{N_{k}-1}}{P_{k}^{tr}}\;$$

where $\tau_{k}\;$ is the transmission probability for each STA in channel *k*, and $N^{k}\;$ is the number of competing Wi-Fi STAs in channel k. According to Bianchi [29], the Wi-Fi network throughput $S_{k}^{Wifi}$ can be formulated as

(4) $$S_{k}^{Wifi}\left(N_{k}\right)=\frac{P_{k}^{tr}P_{k}^{s}E\left[P\right]}{\left(1-P_{k}^{tr}\right)T_{k}^{\sigma }+\;P_{k}^{tr}P_{k}^{s}T_{k}^{s}+\;P_{k}^{tr}\left(1-P_{k}^{s}\right)T_{k}^{c}}$$

where $T_{k}^{s}\;$ is the average duration of a channel detected as busy owing to a successful transmission, $T_{k}^{c}$ is the average duration of a channel detected as busy owing to a collision, *E*[*P*] is the average packet size, and $T_{k}^{\sigma }$ is the empty time slot duration.

In LTE-U networks, Wi-Fi has an opportunity to access the channel only when LTE-U is OFF. Thus, the throughput achieved by Wi-Fi in the shared spectrum when coexisting with LTE-U can be expressed as follows:

(5) $$R_{Wi-Fi}^{k}=\;S_{k}^{Wifi}\left(N_{k}\right)\times \;\Theta_{k}$$

### 3.2. LTE-U DC and CA Model

**Figure 4:**
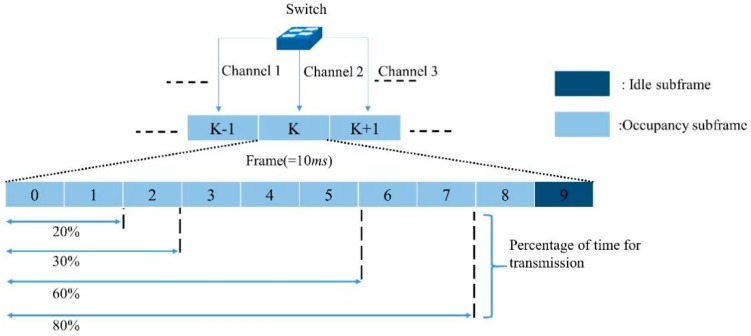
LTE-U DC and CA model.

To devise a duty-cycle based LTE-U transmission, we consider a TDD configuration, as shown in Figure 4. In this structure, an LTE-U frame of 10 ms is divided into multiple subframes while maintaining the same frame length as that of LTE. In Figure 4, the dark blue slots represent blank subframes, which can be used by Wi-Fi systems, with the remaining subframes used by LTE-U systems. Four different duty cycles are considered (20%, 40%, 60%, and 80%), which can be configured by the operators according to the network requirements. As shown, LTE will transmit for (1-Θ) percent of the time from the allocated duty cycle period and will be mute for Θ percent of the time. A channel selection is used to choose the operating carrier by LTE-U BS. Therefore, by choosing the cleanest channel based on the received power measurements, it can be used as a frequency domain coexistence mechanism to guarantee that LTE-U is a “good neighbor” in the unlicensed band [30]. The design of a proper channel allocation functionality can significantly increase the overall efficiency of the LTE-U operation. Specifically, we can see from Equation (1) that the selection of channels will have an impact on the achieved throughput performance mainly through the $\Theta_{k}$ and $SINR_{m_{k}}\mathrm{terms}\;.\;$Thus, if the selected *k*th channel is not used by other cells, a higher throughput will follow. In addition, if the selected *k*th channel is affected by low interference levels, a high $SINR_{m_{k}}$ will be observed along with higher throughputs. Therefore, the channel selection for an LTE-U BS should be able to dynamically identify and capture the key information regarding the present utilization of the channels allowing the most suitable ones to be selected. Therefore, solutions recognizing the best channel are of high interest in exploiting the full potential of LTE-U. However, it is always not the best case. This is because (as environment remains the same) LTE-U will try to remain within the same parameters (i.e., channel and duty cycle) as long as it does not find other parameter that can maximize its cost function. Residing with the same parameters for a long period of time will continuously decrease the access opportunity for the Wi-Fi user of that channel hence decreasing the fairness. Our algorithm intended to address this problem by avoiding an LTE-U to occupy the same channel for a long duration and thus providing more access opportunities for Wi-Fi users under low traffic load condition. Therefore, the proposed Q-learning algorithm helps LTE-U to switch its current transmission from the optimal channel to a second optimal channel easily in runtime without any increase in complexity in the algorithm. In addition to this, Q-learning with its simple modeling approach provides LTE-U BS to learn from the environment and automatically adapt to an appropriate parameter providing robustness to its dynamic and uncertain operating environment.

### 3.3. Q-Learning Based Joint ADC and DCS for LTE-U

Q-learning has been used in various studies to enhance the coexistence mechanisms and use them individually to learn the best possible strategies to achieve the target [31]. In this paper, we use Q learning for monitoring the Wi-Fi STA traffic load on an unlicensed channel and adjust the LTE transmission accordingly. The objective of joint ADC and DCS reinforcement learning is to determine a policy by which the LTE-U BS will choose the channel and duty cycle period based on measurements observed during a muting period. 

Q-learning is a model-free reinforcement-learning algorithm. The Q-learning process is built on a Q-function (*Q* (*k*, *a*)), which is updated when it obtains a reward *r* from a state transition after the agent carries out a certain action *a*. We use the single-state Q-learning approach with a null discount rate [31] given by

(6) Q(k, a)= (1−α)∗Q(k, a)+ α∗r(k, a)

where *α є* (0, 1) is the learning rate and r(k, a) is the reward obtained as a result of the current action. At initialization, *Q*(*k*, *a*) is set to an arbitrary value *Q_init_*_._ Based on the value of *Q*(*k*, *a*), the proposed duty cycle decision making for LTE-BS follows the softmax policy [31]. The softmax approach is popular owing to its effective and popular means of balancing the exploration and exploitation in reinforcement learning. In a softmax policy, the duty cycling action *a* is chosen based on the following probability:

(7) $$p\left(k,a\right)=\frac{exp^{Q\left(k,\;a\right)/Temp_{k}}}{\sum_{\;a^{\prime }=1}^{n}exp{\;}^{Q\left(k,\;a^{\prime }\right)/Temp_{k}}}$$

where $Temp_{k}$ is the temperature function, which helps reduce the temperature as the number of actions generated by LTE-U BS increase. The resulting amount of exploration will be progressively decreased as LTE-U learns the best solution, and can be expressed as follows:

(8) $$Temp_{k}=\frac{Temp_{k}^{init}}{log\left(1+Y_{k}\right)}$$

where $Temp_{k}^{init}$ is the initial temperature and $Y_{k}$ is the action counter. A high temperature causes different actions to be equiprobable whereas a low temperature causes a greater difference in the selection probability for actions that differ in their value estimates. Hence, our scope of Q-learning is to discover the optimal policy for choosing an action in a given state that maximizes the value of the overall reward. To learn this policy, an agent must estimate the value-function through experience. The main components of Q-learning are as follows:

- An agent is the LTE-U BS. LTE-U BS can change its muting time period for each 10-ms duty cycle period;
- An action that an agent can take is a set of duty cycle patterns A = {0.2, 0.4, 0.6, and 0.8}. Herein, a duty-cycle pattern of 0.4 indicates that LTE-U mutes 0.8 portion of its frame time and transmits during the remaining 0.4 portion of 10 ms;
- Q-learning decisions are taken for every duty cycle duration, which is repeated every 10 ms;
- A state indicates the carrier that is selected for operation {1, 2,…, *K*};
- A reward function is a utility function that guarantees the selection of an appropriate duty-cycle action in the best available channel. This means the chosen action will be maintained close to the target duty-cycle value, offering fair coexistence with other co-located systems (Wi-Fi). At the same time, it compares the goodness of the selected channel with other available channels. The reward for action a of an agent is given through the following function:

(9) $$r^{k}=\;\frac{\sigma \;*\;\left(\theta_{k}^{target}\;-│\theta_{k}^{target}-\theta_{k}^{action}│\right)\;}{N_{k}}\;\mathrm{for}\;\theta_{k}^{target}-\theta_{k}^{action}\;>0\;0\;\mathrm{for}\;\theta_{k}^{target}-\theta_{k}^{action}\;<\;0$$

where σ defines the fraction of positive rewards, and $\;\theta_{k}^{target}$ and $\theta_{k}^{action}$ are the predefined optimal duty cycling action and the chosen action values from the set of available duty cycling actions, respectively. In our system, the target duty cycling action in the network is the ratio of the sum of active LTE-U UEs to the total number of active users in the channel, and can be denoted as follows:

(10) $$\theta_{target}^{k}=\frac{M}{M+N_{k}}$$

The proposed Q-learning algorithm is briefly described in Algorithm 1 below:

**Algorithm 1** Q-Learning algorithm for joint ADC and DCS mechanism.1: **Input:** Duty cycle patterns, θ; Number of channel, K; Number of Wi-Fi users in the channel, *N_k_* 2: **Output:** Optimal duty cycles and channels. 3: **Initialization:** Q-table, *Q*(*k*, *a*); Selection probability, *p*(*k*, *a*); Action counter, Y_k_; Learning rate, α; Initial temperature, $Temp_{k}^{init}$; Positive reward, σ; 4: Randomly choose starting state (i.e., next state) 5: Set the iterations = 0 6: **Learning procedure:** 7: **loop**
8:      current state = next state 9:      execute the action a = $\underset{a}{max}\left(p\left(k\;,a\right)\;\right)$ 10:    Receive the immediate reward: 11:    **if** $(\;\theta_{k}^{target}-\theta_{k}^{action}>0$) 12:    $r^{k}=\frac{\sigma \;*\;\left(\theta_{k}^{target}\;-│\theta_{k}^{target}-\theta_{k}^{action}│\right)\;}{N_{k}}$
13:    **else** 14:     0 15:    **end**
16:    Update Q (k, a) according to Equation (6) as follows: 17:    Q(k, a)= (1−α)∗Q(k, a)+ α∗r(k, a) 18:     Update action counter *Y_k_. = Y_k_ + 1.* 19:     Compute the $Temp_{k}\;and\;p\left(k,a\right)$ according to softmax policy according to the Equations (6) and (7). 20:   $Temp_{k}=\frac{Temp_{k}^{init}}{log\left(1+Y_{k}\right)}$; $p\left(k,a\right)=\frac{exp^{Q\left(k,\;a\right)/Temp_{k}}}{\sum_{\;a^{\prime }=1}^{n}exp{\;}^{Q\left(k,a^{\prime }\right)/Temp_{k}}}\;$ 21:     Update p (k, a). 22:     Choose the next state = $\underset{k}{max}\left(Q\left(k\;,a\right)\;\right)$
23:   **end loop** 24: **Monitoring the wireless environment:** 25: **while** (true) **do** 26:    Periodically monitor the wireless environment 27:    **if** (changes is identified) then 28:       Reset *Y_k_*
29:    **end** 30:  **end**

Many LTE-U BSs available on the market are supported using a single or multiple Wi-Fi interface [32,33,34,35] for monitoring the carrier and notification purposes in LTE-U. ULTRON [33], which operates in a LTE-U BS, employs Wi-Fi embedding to transmit Wi-Fi data over an unmodified LTE PHY. In addition, the same method is used to recognize a Wi-Fi preamble transmission directly applying LTE PHY. In addition, ULTRON also facilitates scalable Wi-Fi sensing to efficiently set up a single Wi-Fi sensing interface to jointly enhance the performance of both LTE and Wi-Fi [36]. Hence, we can use existing estimation techniques such as a Kalman filter [37], machine learning techniques [38], and power techniques [39] to approximate the number of active Wi-Fi STAs in each channel using LTE-U [40]. Here, we use a Wi-Fi preamble decoding and energy detection (ED) mechanism in the time domain [41,42] without synchronization to the Wi-Fi STAs. During every muting period, LTE-U BS listens to the carrier to evaluate the collision probability ($P_{k}^{coll}$*)* and channel idle probability ($P_{k}^{idle}$) among Wi-Fi STAs. The total number of observed slot times is denoted as $C_{k}^{listen}$ and the number of collisions in the observed period is represented by $\;C_{k}^{coll}$. Hence, $P_{k}^{coll}$ can be calculated as $P_{k}^{coll}$ = $C_{k}^{coll}$*/*$C_{k}^{listen}$. Furthermore, $P_{k}^{idle}$ can be acquired based on the ratio of the total number of idle slots to the total number of observed periods, i.e., $P_{k}^{idle}$ *=* $C_{k}^{idle}$*/*$C_{k}^{listen}$, where $C_{k}^{idle}$ is the number of idle time slots in channel k.

When an LTE-U BS is muted, the probability of channel *k* being idle is when all Wi-Fi STAs do not transmit is $P_{k}^{idle}=\;{\left(1-\;\tau_{k}\;\right)}^{N_{k}}$, where $\tau_{k}$ represents the transmission probability of Wi-Fi STAs when LTE-U BS is muted. Similarly, the probability that the channel will experience a collision when at least one of the ($N_{k}-$1) remaining station transmits is $\;P_{k}^{coll}=\;{\left(1-\;\tau_{k}\right)}^{\left(N_{k}-1\right)}$. Hence, $P_{k}^{coll}\;=\;1-\frac{P_{k}^{idle}}{1-\tau_{k}}\;$ and numerically solving the equation for $\;\tau_{k}$, we obtain $\tau_{k}=\;1-\frac{P_{k}^{idle}}{1-P_{k}^{coll}}$.

Now, the active number of Wi-Fi STAs is obtained by solving the equation, $N_{k}=\;log_{\left(1-\tau_{k}\right)\;}\;P_{k}^{idle}$, which is $\frac{log\left(P_{k}^{idle}\right)}{log\left(1-\tau_{k}\right)}\;.$

### 3.4. Fairness in Unlicensed Spectrum

LTE-U is deemed successful if its coexistence with Wi-Fi is fair. A fair coexistence approach must provide all coexisting networks with equal opportunities to access the medium. However, this kind of fairness is limited between a system of the same type and a system having similar system parameters. In an LTE-U network scenario, both technologies are diverse, having major design dissimilarities. The normalized throughput achieved by both systems will be a good indicator of the fairness. That is, when presenting LTE-U into a shared channel, the effect on the existing STAs should be similar to that of adding the same type of STAs. Hence, in the remainder of this paper, the Jain fairness index on the achieved throughput is implemented as key performance metric for the fairness evaluation. The normalized throughput for each participating network is achieved based on the ratio of its achieved throughput to the maximum throughput attained during the standalone operation.

(11) $$Jain\;fairness\;index\;\left(\mu_{k}\right)\;=\;\frac{{\left(Th^{wifi}{}_{k}\;+\;Th^{LTE-U}{}_{k}\right)}^{2}}{2[\left((Th^{wifi}{}_{k}\right){\;}^{2}+\;{\left(Th^{LTE-U}{}_{k}\right)}^{2}]}$$

where, $Th^{wifi}{}_{k}\;$and $Th^{LTE-U}{}_{k}$ are the normalized throughputs attained by LTE-U and a Wi-Fi network. 

### 3.5. Efficiency of Spectrum Utilization

Once a fair coexistence of Wi-Fi and LTE-U is accomplished, we can concentrate on an efficient spectrum utilization of the unlicensed spectrum. To obtain the maximum throughput, each time slot should be assigned to the system that makes best use of it. In an LTE-U scenario, the LTE-U achieves a superior performance over Wi-Fi in terms of spectrum utilization owing to the advantage of its scheduler when compared to contention-based access. Hence, to increase the efficiency of the spectrum, LTE-U must be given greater access opportunity. However, this decision can significantly disturb the fairness criteria of a coexisting system. In this study, we combat this tradeoff through switching to most uncongested or free channels. The achieved throughput performance greatly relies on the number of users connected to the channel and the interference. Thus, operating in the cleanest channel provides more access opportunities for LTE-U users, increasing the throughput performance of the network. In addition, it helps Wi-Fi STAs deliver more access opportunities under low load conditions by deterring an LTE-U BS from keeping a channel busy for a longer period, i.e., if an LTE-U UE cannot finish its transmission during its ON period, it will not stay on the same channel to transmit during the next frame. Instead, it switches its existing transmission from the present channel to any other free channel. By doing so, Wi-Fi STAs in the current channel will acquire the opportunity to access the channel. Moreover, by switching the LTE-U channel after its transmission, the muting period of the LTE-U is highly reduced, contributing to an increase in spectrum utilization. We calculate the network efficiency as the ratio of total achieved network throughput by the proposed method to the maximum achievable network throughput $\left(Th_{\max\;}^{k}\right)$ as follows:

(12) $$Network\;efficiency\;\left(\eta_{k}\right)=\;\frac{Th^{wifi}{}_{k}\;+\;Th^{LTE-U}{}_{k}}{Th_{max}^{k}}$$

## 4. Performance Evaluation

In this study, we developed an LTE-U system level simulation platform using MATLAB. The system simulation statistics were obtained by acquiring the mean values over random user positions. A set of unlicensed channels and APs were deployed in each channel with a variable number of STAs. The LTE-U BS is permitted to switch over unlicensed carriers and notify the LTE-U UE to complete the communication procedure in the corresponding channel through a licensed carrier. The other simulation parameters for LTE-U and Wi-Fi are set as shown in Table 1.

For an evaluation, the proposed algorithm is compared with a duty-cycle based method and a COT-based channel selection approach. In the duty-cycle based approach, which is similar to those described in [8,9], and [19], LTE-U BS selects the single best channel and applies the Q-learning approach for a dynamic duty cycle selection. Using the COT-based channel selection approach [16,42], the LTE-U BS adjusts its ON time, according to the channel occupancy measurement of coexisting Wi-Fi users, i.e., the LTE-U ON time is proportional to the measured idle time in the channel. LTE-U BS switches the channel with the highest occupancy time.

**Figure 5:**
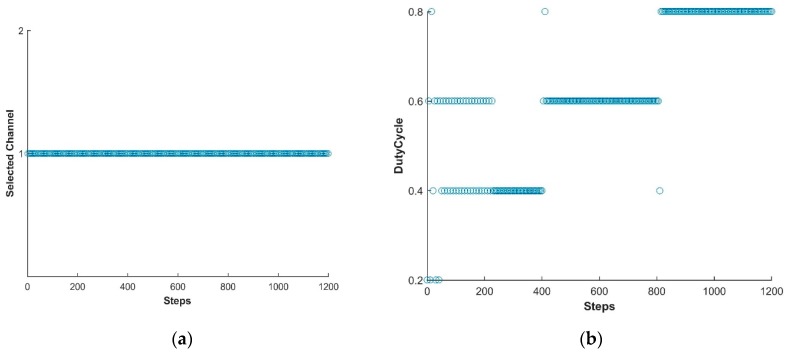
LTE-U BS with DC only method. (**a**) Channel selection vs number of steps; (**b**) Dutycycle selection vs number of steps.

**Figure 6:**
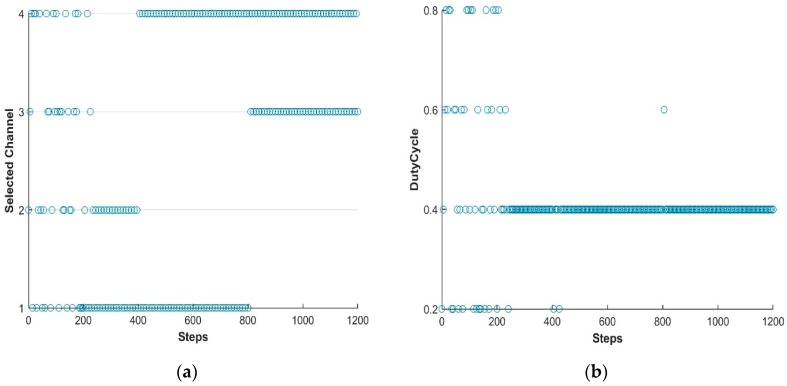
LTE-U BS with COT method. (**a**) Channel selection vs number of steps; (**b**) Dutycycle selection vs number of steps.

**Figure 7:**
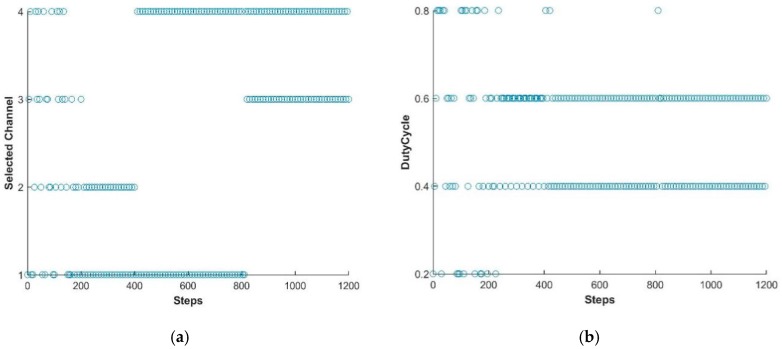
LTE-U BS with proposed method. (**a**) Channel selection vs number of steps; (**b**) Dutycycle selection vs number of steps.

In this study, the LTE-U BS is facilitated using the Q learning approach [31,44] and thus the BS is capable of adjusting its available actions according to the change in environment. In this way, LTE-U BS can modify the Q-matrix and acquire new best duty-cycle patterns and achieve its goal. Figure 5, Figure 6 and Figure 7 show the simulation results of the joint LTE-U DCS and ACS mechanisms for DC only, COT, and the proposed mechanism. Here, the number of active STAs of each channel changes every 400 steps. Ten STAs of each channel move to other channels, maintaining the same number of total STAs in the network. In Figure 5a, we can see that the DC-only scheme uses a single channel throughout the simulation. However, in Figure 6a and Figure 7a, we can see that COT and the proposed scheme switch to best channel based on the changes in the number of STAs in the channel. Likewise, Figure 5b, Figure 6b, and Figure 7b, show the LTE-U BS duty-cycle pattern against the number of steps. In Figure 5b, the DC-only scheme adaptably maintains its muting period according to the change in the number of users in the operating channel. However, in Figure 6b and Figure 7b, we can see different trends in the LTE-U BS duty cycle against the number of steps, the reason being that the COT and the proposed scheme both have easier channel switching features. They switch to the most optimal channel and maintain the highest duty cycle pattern according to the target value. 

**Figure 8:**
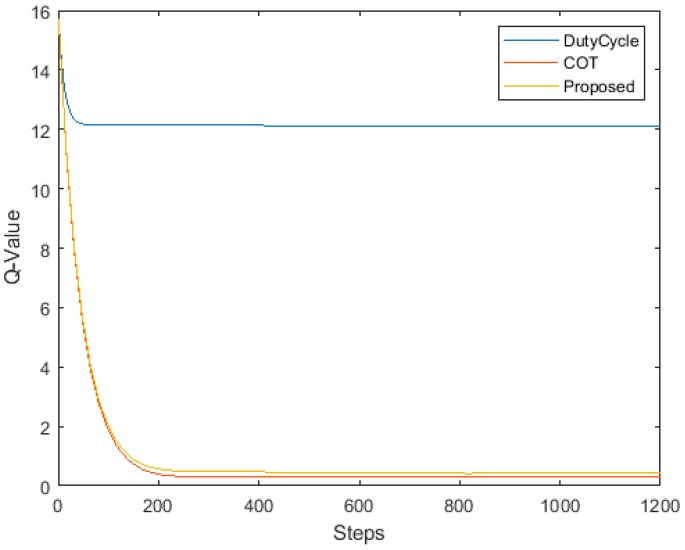
Q-value convergence.

Figure 8 shows the convergence of the Q-learning process by the LTE-U BS. The horizontal axis indicates the number of steps, and the vertical axis is the aggregated sum of values of the Q matrix, i.e., the Q-value. When the Q matrix converges, the LTE-U BS has learned the present configurations and can execute the optimal duty-cycle pattern in any channel. We can see that the sum of the Q-matrix decreases during the start of the learning process. This occurs because the LTE-U BS tries to explore many different states in search of achieving the highest reward. 

As the learning continues, the LTE-U BS discovers the channel and duty-cycle pattern that can deliver the highest fairness and effective coexistence with a Wi-Fi network, increasing the rewards received. As indicated in Figure 8, after approximately 240 iterations, LTE-BS determines the configurations that can direct the system toward a fair and optimal coexistence. By contrast, the DC-only scheme converges must faster than the other schemes because it has only one state. The convergence is directly proportional to the available $\;{\left(no.of\;state\right)}^{no.of\;actions}$. After the system has already learned any changes in the environment, the configuration requires much fewer steps (approximately less than 20 iterations) to converge. 

**Figure 9:**
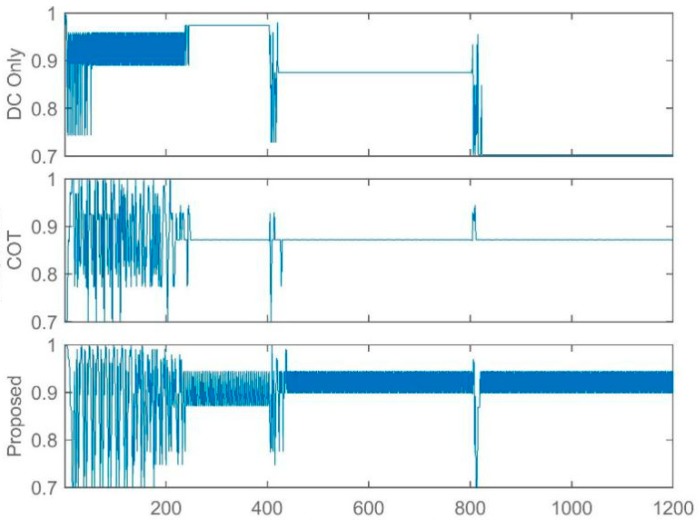
Fairness versus number of steps.

Figure 9 shows the fairness index achieved in the proposed network scenario with the DC-only, COT, and proposed schemes. The simulation results indicate that the proposed scheme attains the highest fairness index in a dynamic network environment when compared to the other two schemes. This is because the proposed scheme attempts to maintain its duty cycle close to the target value, offering a proportional duty cycle configuration according to the load of each coexisting network. However, the COT-based scheme attempts to maintain its target value toward the idle time of the operating channel. In the COT-based scheme, the LTE-U continuously suffers from both airtime and throughput unfairness as the number of users increases. The DC-based scheme achieves the highest fairness index in a constant environment because it only operates in the single best channel. Although the DC-only mechanism learns to adapt its duty cycle according to a changing environment, as shown in Figure 5b, the fairness of the network degrades because there is always the possibility of another channel delivering a higher fairness to the network.

**Figure 10:**
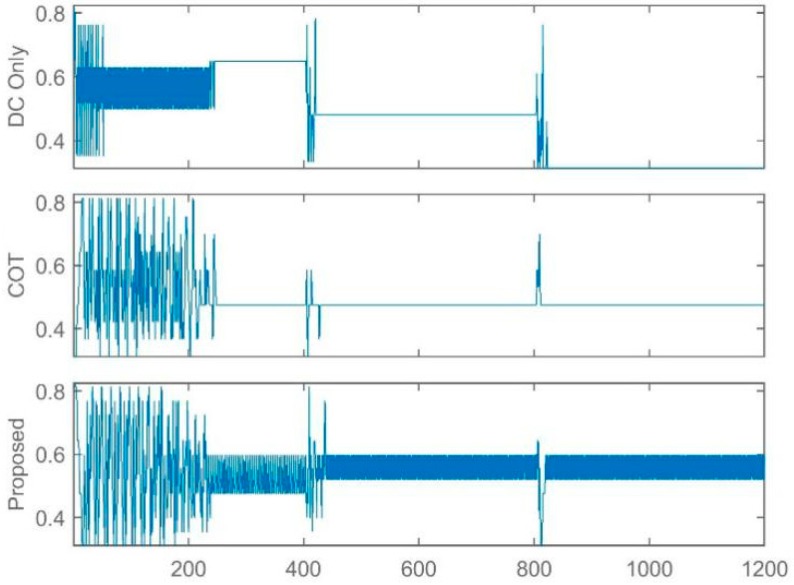
Efficiency versus number of steps.

Figure 10 shows the network efficiency achieved in a network scenario when applying the DC-only, COT, and proposed schemes. The maximum network efficiency occurs under a scenario in which LTE-U uses a single dedicated channel among the available channels (one channel used by LTE-U and three other channels used by Wi-Fi) without having to share with Wi-Fi. The simulation results in Figure 10 indicate that the proposed scheme achieves the highest overall network performance compared to the COT-based and DC-only schemes. This is because our proposed scheme provides more airtime access opportunity to LTE-U than the COT-based approach (higher priority given to Wi-Fi), allowing it to operate in the most uncongested channel. Because the DC-only approach operates in the single best channel for both fairness and efficiency, it demonstrates the highest performance in a constant environment. However, with a change in environment, its performance is significantly reduced because it lacks a switching feature to operate on the most uncongested channel in the network. 

## 5. Conclusions

The use of LTE-U in an unlicensed spectrum is an auspicious alternative to satisfy the multimedia data demand foreseen by upcoming IoT systems. For coexistence between LTE-U and Wi-Fi systems, fair coexistence and a maximization of the channel utilizations are two important design goals. However, achieving both of these design goals together is extremely difficult. This study presented a Q-learning based joint adaptive duty cycling (ADC) mechanism and a dynamic channel switch (DCS) mechanism to facilitate a fair and efficient coexistence. The adaptive DC mechanism supports a fair channel access opportunity by muting a certain number of subframes for Wi-Fi STAs, whereas the DCS mechanism offers more access opportunities for LTE-U UEs by avoiding the UEs to occupy a crowded channel. The simulation results demonstrate a fair coexistence and reveal the benefit of using the proposed mechanism over other DC-only and COT-based mechanisms.

## Figures and Tables

**Table 1:** Simulation parameters.

Parameter	Value
**Common Parameters:**	
Number Of channel	4
Simulation Time	1200 ms
Bandwidth	20 MHz
Spectrum	5 GHz
Traffic Model	Full Buffer
Transmission Scheme	OFDM
**LTE-U Parameters:**	
LTE-U BS	1
UE Number	10
Frame Duration	10 ms
Duty Cycle	0.2/0.4/0.6/0.8
Transmit Power	15 dBm
Terminal Noise Figure	9 dB
PL Model	32.8 + 20*log10(f) + 16.9*log10(d) (ITU InH model [43])
Discount Factor β	0
Learning Factor α	0.3
T_init_	0.5
**Wi-Fi Parameters:**	
Wi-Fi AP	4
STA Number	10/20/30/40
Wi-Fi MAC Protocol	DCF
Time Slot	50 µs
CW	32–256
SIFS	28 µs
DIFS	128 µs
